# A Landslide Monitoring System for Natural Terrain in Korea: Development and Application in Hazard Evaluations

**DOI:** 10.3390/s21093040

**Published:** 2021-04-26

**Authors:** Young-Suk Song, Byung-Gon Chae, Kyeong-Su Kim, Joon-Young Park, Hyun-Joo Oh, Sueng-Won Jeong

**Affiliations:** 1 Geologic Environment Division, Korea Institute of Geoscience and Mineral Resources, Daejeon 34132, Korea; yssong@kigam.re.kr (Y.-S.S.); kks@kigam.re.kr (K.-S.K.); ohj@kigam.re.kr (H.-J.O.); swjeong@kigam.re.kr (S.-W.J.); 2 Policy and Planning Division, Korea Institute of Geoscience and Mineral Resources, Daejeon 34132, Korea; bgchae@kigam.re.kr

**Keywords:** shallow landslides, monitoring system, natural terrain, infinite slope stability, hazard evaluation

## Abstract

This study describes the development of a landslide monitoring system for the purpose of reducing damages caused by landslides in natural terrain. The system was developed to analyze the effects of landslide-inducing rainfall and the behavior of slopes through 12 monitoring stations that are distributed across eight national parks in Korea. Several sensors and a data acquisition equipment to monitor landslide were installed in each station. The composition of the system and its operating program were designed to efficiently manage the sizeable amounts of real-time monitoring data that are collected from the various stations. To test the potential of the developed system for reliable landslide hazard evaluations, data measured over a five-year period by the two monitoring stations in Jirisan National Park were analyzed. Subsequently, the suction stress of the soil over the monitoring period was calculated by applying laboratory test result of the geotechnical and unsaturated soil properties in the analysis domain area. The infinite slope stability analysis combined with an effective stress concept based on the suction stress was applied to calculate the factor of safety. This method also enabled the temporal and quantitative evaluation of slope stability in natural terrain. In addition, based on the monitoring and slope stability analysis results, an analysis for the spatial classification of landslide hazards was conducted. The analysis results quantitatively and statistically demonstrated that 98% of historical landslide initiation areas were classified as high hazard levels.

## 1. Introduction

Many areas are becoming exposed to multiple landslide hazards due to extreme rainfall conditions. Global climate change has introduced significantly higher rainfall intensity and cumulative rainfall than those from past records in Korea. This has directly resulted in greater casualties and property damage than in the past [1,2,3]. Thus, there is growing social demand for measures against landslide damage resulting from extreme rainfall. In addition, with the recent advancements in information, communication, and computing technology, an increasing number of studies are aiming to develop technologies that can analyze landslide threats according to changes in rainfall in real time, which can allow for early warning systems that can fundamentally reduce potential damage on mobile subjects [4,5,6,7].

The prediction of where landslides occur requires the evaluations of spatially varying rainfall conditions and the intrinsic factors of natural slopes, such as topographic, geological, and geotechnical properties. However, to predict when landslides occur, one must understand how such conditions and properties vary over time. Namely, it is vital to determine how the hydro-mechanical properties within slope soil mass change over time as rainfall infiltrates into the soil, which helps clearly understand critical conditions leading to the moment of actual landslide events. For this purpose, researchers must go further than analyzing fixed and intrinsic factors and observe in real time the changes in slope soil mass properties according to variations in rainfall conditions. To achieve this task, real-time monitoring of natural slopes is essential.

To detect landslide occurrences in advance, it is necessary to observe rainfall conditions and the resulting response characteristics within slopes. Based on the observations, the changes in rainfall and soil conditions upon landslide occurrence should be analyzed. For instance, some studies adopted modeling approaches based on either experiments [8] or statistical methods [9,10] to interpret soil erosion and shallow landslide phenomena. On the other hand, in contrast to the multitude of monitoring-related studies on artificial slope stability that have been conducted in Korea [11,12,13,14,15,16], very few studies have focused on monitoring the changes in soil properties in natural slopes.

One of the most crucial element of landslide early warning technology for natural slopes is in-situ monitoring in areas with the potential for landslide occurrence; it is related to comprehensive measurements of various geological and geotechnical factors related to landslides. With this as a basis, landslide monitoring systems for natural slopes should be capable of detecting the potential of landslide occurrence in advance and issue alerts to local citizens.

Currently, the Korea Institute of Geoscience and Mineral Resources (hereafter referred to as KIGAM) is in the process of establishing and operating LAndslide MOnitoring Systems (hereafter referred to as KIGAM-LAMOS) for natural slopes in national parks in Korea. While numerous state-of-the-art landslide monitoring systems have been oriented to detecting long-term mass displacements using various remote sensing techniques [17,18,19,20], the proposed system is based on contacting sensors to directly detect geo-hydrological and dynamic signals of shallow landslides.

To fully understand the changes in rainfall and soil properties leading to the moment of landslide occurrence, the relevant in-situ measurements should first be conducted, followed by in-depth consideration and accurate judgement. This paper first presents a technical note describing the principles, development, operational architecture, and techniques of KIGAM-LAMOS that mainly aims to monitor geo-hydraulic processes leading to the initiations of shallow landslides. Subsequently, the results of landslide hazard evaluation analyses based on monitored data are provided and discussed to verify the effectiveness of the developed system in determining the landslide potential. The monitored parameters consist of rainfall and matric suction that were measured at two monitoring stations in Jirisan National Park from 2015 to 2019. Using the five-year monitoring data, the analyses were conducted in the following two interconnected but independent categories: (1) temporal evaluations of suction stress of soil and slope stability; (2) spatial classifications of landslide hazards for the analysis domain area. To verify the effectiveness of the spatial classification results, a comparative analysis was performed with historical landslide areas that were found to have occurred in the analysis domain area.

## 2. Landslide Monitoring System for Natural Terrain: KIGAM-LAMOS

### 2.1. Concept and Configurations

In general, rainfall-induced landslides are initiated in the form of slope failure caused by an increase in pore water pressure as the groundwater level rises or wetting front advances in soils. However, Fredlund and Rahardjo [21] reported that the elevation of groundwater that is deep below the surface does not significantly affect shallow slope failure, even under intense rainfall. In the case of natural slopes, shallow failures are generally caused by the wetting front advancement resulting from rainfall infiltration instead of groundwater level rises [22,23]. Due to rainfall infiltration, unsaturated soil above the groundwater level becomes increasingly saturated, resulting in a decrease in negative pore water pressure. This results in a decrease in effective stress due to changing suction stress levels in the unsaturated soil, which affects the stability of unsaturated slopes [22,24]. For this reason, several studies are underway to monitor unsaturated properties for natural slope stability evaluation [25,26,27,28,29]. Shallow landslides, a typical type in Korea, occur within depths of more or less 1 m [30]. As slope failures occur due to changing water content in natural slopes under rainfall, it is necessary to consider the effective stress at different depths [31,32,33,34].

Therefore, the KIGAM-LAMOS monitoring sensors were installed in a manner to measure changes in effective stress at various depths during rainfall that may contribute to the triggering of shallow landslides in natural terrain. The effective stress in an unsaturated state of soil can be calculated by measuring both volumetric water content and matric suction. Accordingly, sensors that are suitable for natural slopes in Korea were selected based on previous unsaturated slope monitoring studies and experience. In addition, a data acquisition system capable of wireless communication was established to enable real-time data transmission.

**Figure 1:**
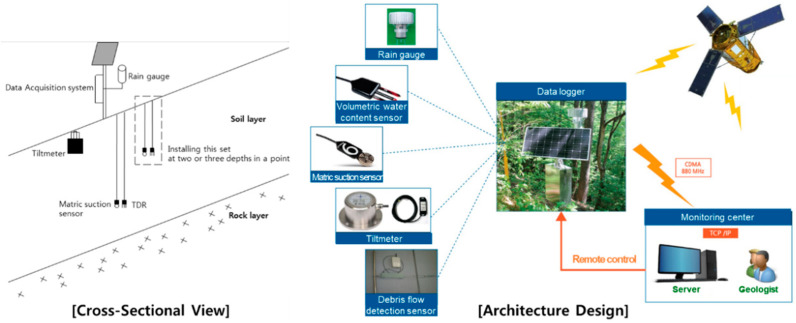
Configuration of KIGAM-LAMOS at a particular monitoring station.

The diagrams in Figure 1 illustrate the cross-sectional view and architecture design of KIGAM-LAMOS at a particular monitoring station. As shown in the figure, volumetric water content and matric suction were measured at various depths to measure the descent of the wetting front resulting from rainfall infiltration in addition to the changes in saturation. Considering that the general depth of natural slope soil layers in Korea is within 2 m, the sensors were installed at depths of 0.5 m, 1.0 m, and 1.5 m below the ground surface.

Decagon 5TM sensors from ICT International (Armidale NSW 2350, Australia) were installed to observe changes in volumetric water content in the direction of gravity as rainfall infiltrates into the soil layers from the surface. As rainfall infiltrates into the soil, the wetting front descends from the soil surface, and in this process, saturation takes place as a wetting band is formed. The expansion of the wetting band causes shallow failure in the upper soil layer. To measure matric suction, MPS-6 sensors from Decagon were installed. The upper soil layers of natural slopes exist in an unsaturated state, and as rainfall infiltrates, the changing saturation state results in a change in suction stress that eventually leads to failure in the upper soil layer.

On the other hand, tiltmeters, specifically MV-5B sensors from NGI (Seoul, Korea), were installed to measure the displacement of natural slopes. In addition, rain gauges were installed to measure the rainfall amount and intensity of the monitoring area. WDR-205 sensors from Wedaen (Seoul, Korea) were used for the rain gauges, which are based on the tipping bucket method.

An electronic wire sensor (EDZ-004, Sakada Denki, Tokyo, Japan) was installed at a lower point of each monitoring station to detect movements and measure velocities of debris flows. An electronic signal is induced and transmitted to a data logger as the wire is cut by loads larger than 150 kgf.

Table 1 lists the key specifications of the sensors installed in the landslide monitoring system.

### 2.2. Selection of Landslide Monitoring Station Locations

In the stage of deciding the locations for landslide monitoring stations, the potential for landslide occurrence should be considered above others. As such, various factors related to landslides should be comprehensively determined. From this perspective, geological conditions should be considered with the highest priority when selecting sites for landslide monitoring. Locations that exhibit representative geological conditions of Korea with a distribution of granite, gneiss, and sedimentary rocks were selected. In addition, the following types of areas were considered with higher priority: areas with suitable conditions (in terms of location and topography, etc.) for the installation of monitoring systems; areas that allow for the sustainable and efficient maintenance of monitoring systems after installation; history of large-scale landslides; the influence areas and paths of typhoons and heavy rainfall, which are key causes of landslides; factors that influence landslides such as soil layers and soil distribution characteristics; the risks of human casualties and property damage in the event of landslides. Furthermore, the ease of licensing for the installation of landslide monitoring systems, convenience in terms of long-term operation and maintenance of monitoring systems, and soil and forest type distributions/terrain conditions that allow for various field tests (in tandem with landslide monitoring) were also considered.

Candidate installation locations were selected by comprehensively considering the aforementioned factors. The final installation locations were selected after conducting site surveys and considering the installation conditions of each candidate location.

### 2.3. Current Status of Landslide Monitoring Stations

**Figure 2:**
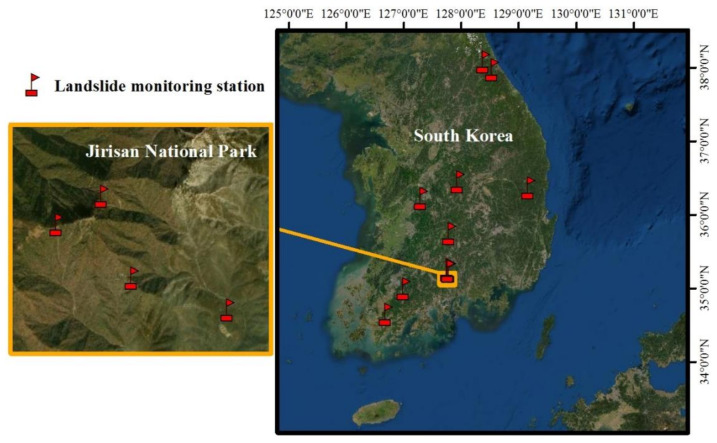
Current status of twelve KIGAM-LAMOS landslide monitoring stations in South Korea.

Korea National Park Service and KIGAM have been engaged in a mutual working-level cooperation to mitigate natural disasters since the memorandum of understanding was signed in November 2014. Based on this cooperation between the two organizations and following the aforementioned selection process for the landslide monitoring station locations, a total of 12 landslide monitoring stations have been installed across eight national parks in Korea thus far. Figure 2 illustrates the locations of the landslide monitoring stations installed in national parks, and Table 2 lists the coordinates of each monitoring station. The mountain national parks where monitoring stations were installed are Seoraksan, Juwangsan, Songnisan, Gyeryongsan, Deogyusan, Mudeungsan, Wolchulsan, and Jirisan. A total of 12 monitoring stations are installed across the national parks: more specifically, the national parks of Juwangsan, Songnisan, Gyeryongsan, Deogyusan, Mudeungsan, and Wolchulsan each have one installed monitoring station, Seoraksan National Park has two monitoring stations (Baekdamsa Temple, Huiungak), and Jirisan National Park has four monitoring stations (Jeseokbong, Jungbong, Rotary Shelter, Jungsalli).

**Figure 3:**
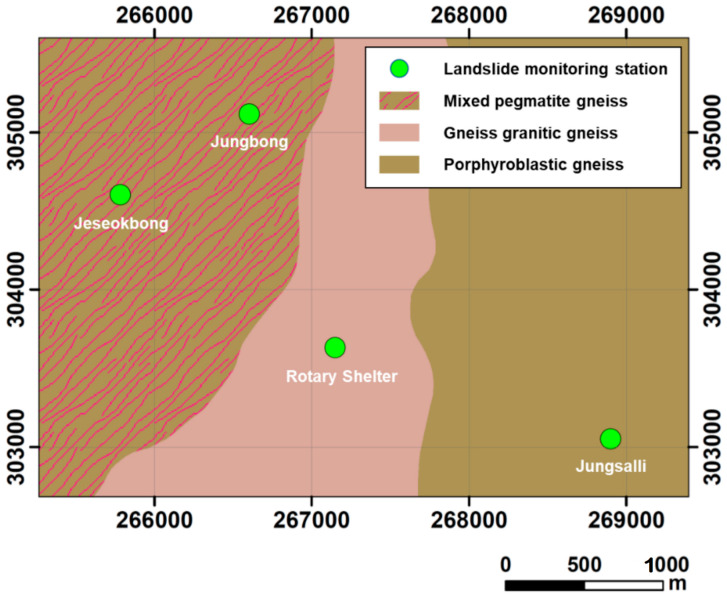
Geological conditions of four landslide monitoring stations in Jirisan National Park.

**Figure 4:**
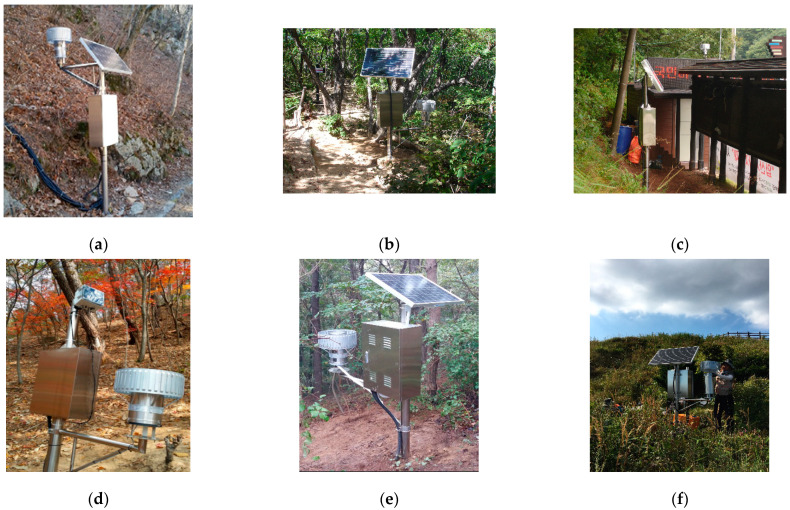

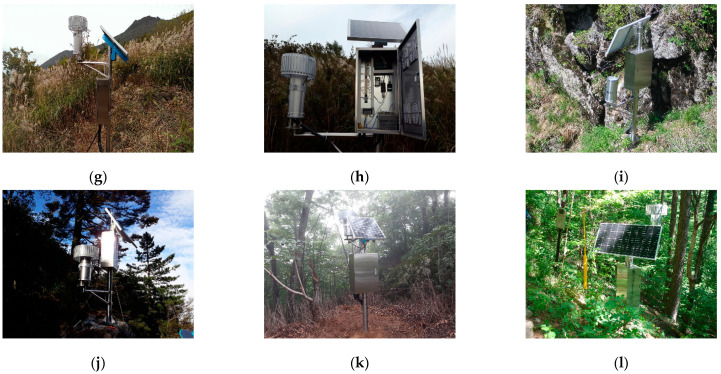
Twelve landslide monitoring stations installed across eight national parks: (**a**) Seoraksan (Baekdamsa); (**b**) Seoraksan (Huiungak); (**c**) Juwangsan; (**d**) Songnisan; (**e**) Gyeryongsan; (**f**) Deogyusan; (**g**) Mudeungsan; (**h**) Wolchulsan; (**i**) Jirisan (Jeseokbong); (**j**) Jirisan (Jungbong); (**k**) Jirisan (Rotary Shelter); (**l**) Jirisan (Jungsalli).

Table 3 shows the geological conditions of the landslide monitoring station locations; especially, three different types of gneiss rocks are underlying weathered soils in Jirisan National Park as illustrated in Figure 3. Table 4 describes the monitoring sensors installed at the stations. As shown in the latter table, the 12 aforementioned stations have a total of 160 water content sensors, 160 matric suction sensors, 24 ground displacement sensors, 8 wire sensors, 12 rain gauges, and 12 data loggers. Figure 4 shows photographs of the main loggers of each of the 12 installed monitoring stations.

### 2.4. Operational Architecture of KIGAM-LAMOS

**Figure 5:**
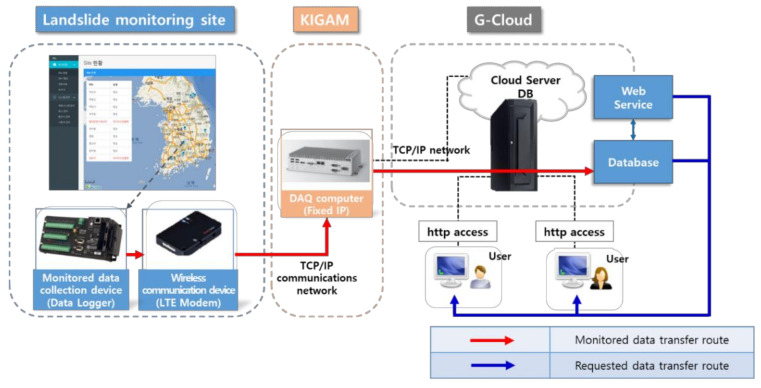
Architecture of data collection and transfer in KIGAM-LAMOS.

A cloud server is useful as it enables administrators and developers to continuously operate the system with greater convenience and efficiency. This is due to the fact that a cloud server remotely provides computing resources to the user, ensuring efficient use of resources. In addition, it allows for the stable storage and supply of monitoring data by gathering scattered resources to a central hub, removing the need for complex management by individual users. Based on such advantages, a cloud server was established as shown in Figure 5 to ensure stable system services and to reduce the costs of server installation and maintenance. The data collected at the landslide monitoring stations are transferred in the following order: the installed sensors, monitoring data loggers, wireless communication devices (LTE modem), a data acquisition (hereafter referred to as DAQ) computer, and a G-cloud server. Users can access the data stored in the server through a web service of KIGAM-LAMOS.

The detailed transfer route of the data is as follows: the measurement values (rainfall amount, matric suction, ground displacement, and water content) obtained from the 12 landslide monitoring stations and the sensor status information (logger battery level, water content, and suction stress) are stored in real time by the data logger and are transferred via an LTE modem to a DAQ computer installed in KIGAM-LAMOS for storage, as shown in Figure 5. The 3G (CDMA) communication network that was initially installed in the 12 monitoring stations was replaced with 4G (LTE) communication in 2017 to change the communication environment and ensure smooth communication with the monitoring system. The DAQ computer is a device that digitizes and analyzes analog signals from data loggers. The final data are stored in a secure cloud server for public institutions.

As the data of landslide monitoring instruments are transferred via a virtual server port, industrial PCs require a static IP to access the data. In addition, as web services are only available with a static web address during monitoring, and as an address is required for monitoring, the cloud server was set with a static IP. As the current LTE modem was assigned with a dynamic IP, filtering with a firewall is not possible, and as the LTE modem itself cannot connect to a VPN, the data were obtained using an industrial PC. The system was configured to allow data to be stored in the DB by establishing a VPN between an industrial PC and the cloud server.

## 3. Landslide Hazard Evaluations Based on KIGAM-LAMOS

### 3.1. Study Area

**Figure 6:**
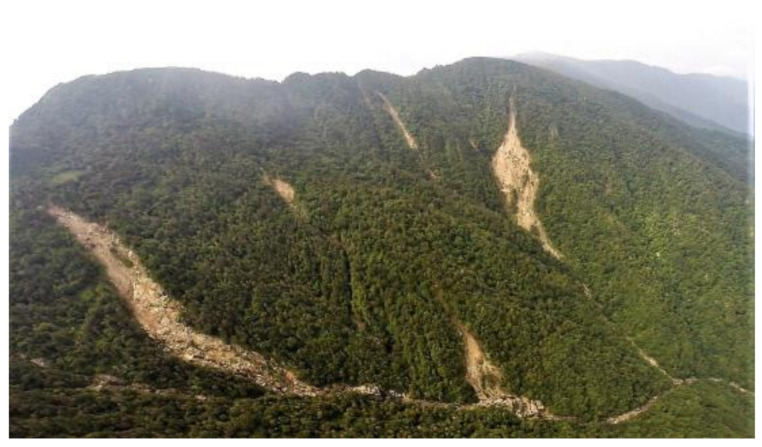
Landslide events that occurred in the Jirisan Jeseokbong area in August 2014.

For a case study to test the proposed KIGAM-LAMOS in terms of its capability in detecting the potential of landslide hazards, measurement data from two selected landslide monitoring stations in Jirisan National Park (Jeseokbong and Jungsalli) were used. The analysis was performed for a five-year period from 2015 to 2019. The area surrounding the Jeseokbong station has steep slopes from the peak of Jirisan Mountain (1915 m above sea level) and consists of a shallow soil layer with depths of up to 2 m covering the bedrock. The area has a robust history of landslide occurrences, and is located on the path of typhoons as they first pass over the Korean Peninsula from the south. Such typhoons pass over this area with a substantial amount of energy, bringing heavy rainfall and strong winds, which results in multiple large-scale landslides and debris flows. In the event of a landslide in this area, the hiking trails that extend from the lower areas to Cheonwangbong Peak are affected by debris flows, causing direct damage to various facilities. In particular, as shown in Figure 6, a large number of large-scale landslides occurred in the Jeseokbong area near the upper parts of the mountain in August 2014 due to heavy rainfall.

**Figure 7:**
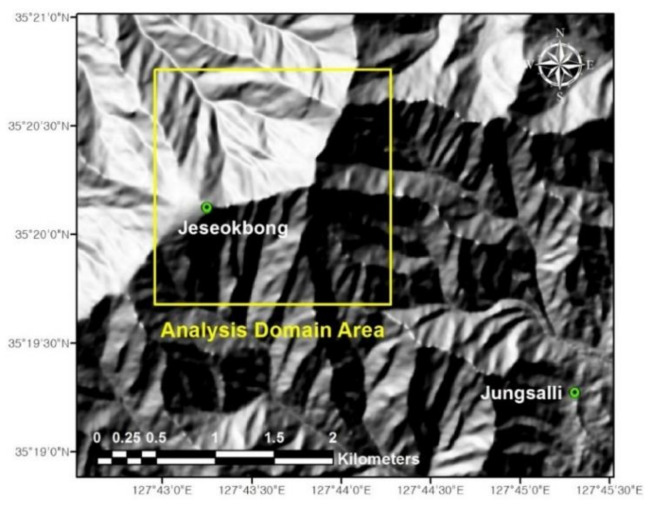
Landslide monitoring stations in Jirisan National Park and the analysis domain area.

Figure 7 shows the selected analysis domain area: a 2 km × 2 km area around Jeseokbong that is prone to frequent landslides. The analysis domain area was assumed to have spatially uniform geotechnical and geo-hydraulic properties throughout, and slope stability analyses to evaluate landslide hazards were conducted based on the five-year monitoring data.

### 3.2. Landslide Monitoring Stations

In addition to the monitoring data from the Jeseokbong station in the analysis domain area, data from the nearby Jungsalli station were also used in this study for analysis. In contrast to the other three stations in Jirisan that use solar energy for power, the Jungsalli station has a stable power supply through cables; this allows the Jungsalli station to transfer and store real-time measurement data with relative stability. Therefore, it is possible to supplement data that are missing in the Jeseokbong station data using the measurement data from the Jungsalli station.

**Figure 8:**
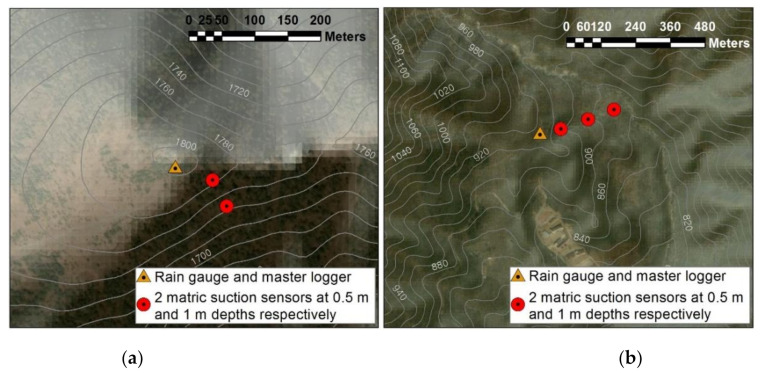
Overview of the Jirisan landslide monitoring sensor layout: (**a**) Jeseokbong station; (**b**) Jungsalli station.

Figure 8 illustrates how the sensors are installed in the landslide monitoring stations. An investigation showed that shallow landslides that occur in natural slopes in Korea are due to shear failures at depths within 1 m from the ground surface [30]. Therefore, the sensors were installed at depths of 0.5 m and 1 m from the ground surface at each of the two stations to measure the change in matric suction at each depth. Figure 8a shows two terrain points where matric suction sensors are buried at the Jeseokbong Station. At each point, two matric suction sensors are buried at depths of 0.5 m and 1 m, respectively; thus, a total of four matric suction sensors are arranged at the Jeseokbong Station. Conversely, the Jungsalli Station configured three aligned terrain points where matric suction sensors are buried, as shown in Figure 8b, and the depths of the sensors are identical to that of the Jeseokbong Station; therefore, a total of six matric suction sensors are arranged at the Jeseokbong Station.

### 3.3. Theoretical Background for Slope Stability Evaluation

To apply the real-time monitoring data to the slope stability analysis, the suction stress of the unsaturated soil according to the wet condition of the slope was considered. Lu and Likos [35] expanded upon the effective stress equations of Terzaghi [36] and Bishop [37] to propose Equation (1), which expresses the stress state of unsaturated soil. This equation considers various phenomena in unsaturated soils to calculate the effective stress of unsaturated soils, and is known to produce relatively accurate suction stress results. As suction stress is closely related to matric suction and saturation, it can be used as an index to evaluate the unsaturated stress state of unsaturated soils. By substituting the equation of van Genuchten [38] into Equation (2), the suction stress calculation formula expressed by Equation (3) can be obtained:

(1) $$\sigma^{\prime }=\left(\sigma -u_{a}\right)-\sigma^{s}=\left(\sigma -u_{a}\right)+\left(u_{a}-u_{w}\right)S_{e}$$

(2) $$\sigma^{s}=-S_{e}\left(u_{a}-u_{w}\right)$$

(3) $$\sigma^{s}=-\frac{\left(u_{a}-u_{w}\right)}{{\left[1+{\left\{a\left(u_{a}-u_{w}\right)\right\}}^{n}\right]}^{m}}$$

Here, *σ*′ is effective normal stress; *σ* is normal stress; *σ^s^* is suction stress; *u_a_* is pore air pressure; *u_w_* is pore water pressure; (*u_a_* − *u_w_*) is matric suction; *S_e_* is effective saturation; and *α*, *n*, *m* are the fitting coefficients of the soil-water characteristic curve of the van Genuchten [38] equation.

**Figure 9:**
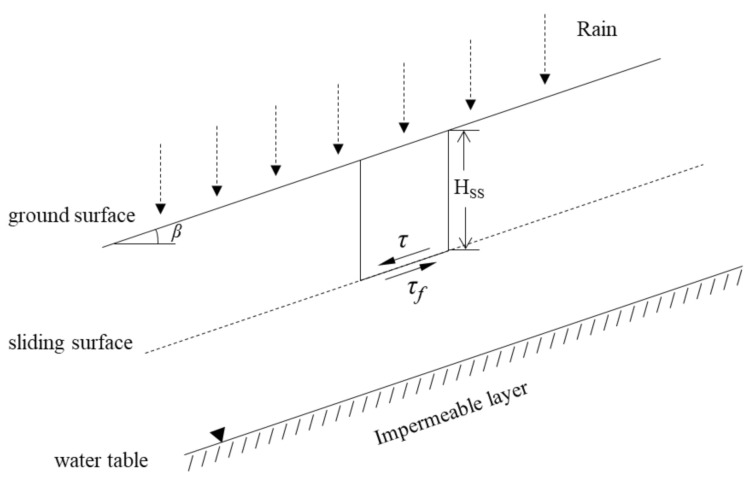
Cross-sectional diagram of infinite slope stability analysis.

In recent years, Lu and Godt [22] proposed to integrate the general concept of effective stress in saturated and unsaturated soil conditions for stability analysis of infinite slope failures at shallow depths due to rainfall. To explain this idea, a cross-section of a general infinite slope is assumed, as shown in Figure 9. Here, *H_ss_* is the depth from the ground surface to the sliding surface, *β* is the slope angle, *τ* is shear stress, and *τ_f_* is shear strength. Based on the diagram, the factor of safety equation for general infinite slopes can be expressed as Equation (4):

(4) $$F_{s}=\frac{\tau_{f}}{\tau }=\frac{c^{\prime }+\sigma^{\prime }\tan\phi^{\prime }}{\gamma H_{ss}\sin\beta \cos\beta }$$

Here, *c*′ is effective cohesion, *σ*′ is effective vertical stress, *φ*′ is effective angle of internal friction, and *γ* is the unit weight of the sliding mass.

By substituting Equation (1), which expresses effective stress considering the aforementioned suction stress into Equation (4), Equation (5) can be obtained:

(5) $$F_{s}=\frac{c^{\prime }+\left[\gamma H_{ss}\cos^{2}\beta -\sigma^{s}\right]\tan\phi^{\prime }}{\gamma H_{ss}\sin\beta \cos\beta }=\frac{\tan\phi^{\prime }}{\tan\beta }+\frac{2c^{\prime }}{\gamma H_{ss}\sin2\beta }-\frac{\sigma^{s}}{\gamma H_{ss}}\left(\tan\beta +\cot\beta \right)\tan\phi^{\prime }$$

As shown in Equation (5), the infinite slope factor of safety equation that considers suction stress can be divided into angle of internal friction, cohesion, and suction stress elements.

### 3.4. Geotechnical and Unsaturated Soil Properties of the Study Area

**Figure 10:**
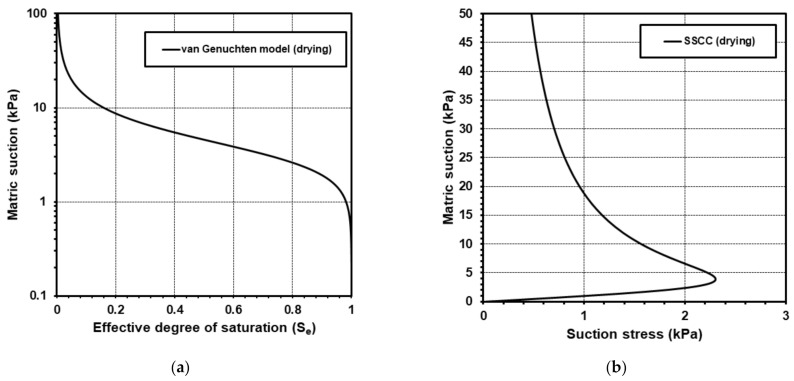
Unsaturated soil properties of the analysis domain area: (**a**) Soil-water characteristic curve; (**b**) Suction stress profile.

Field surveys, sampling, and various soil and unsaturated property tests were conducted to produce data for input parameters required for slope stability analysis of the analysis domain area. Based on the test results and the assumption of near-homogeneous geotechnical properties within the area, input parameter data required for slope stability analysis were determined as shown in Table 5. Based on the pressure plate method of measuring matric suction and pore water content, a soil-water characteristic curve was obtained using the van Genuchten [38] model; curve fitting coefficients such as *α*, *n*, and *m* of Equation (3) were derived. Figure 10a shows the soil-water characteristic curve of the analysis domain area. By applying Equation (3), the suction stress profile according to matric suction can be plotted as shown in Figure 10b. The derived curve has the same shape as the typical suction stress profile of sand proposed by Lu and Likos [39].

**Figure 11:**
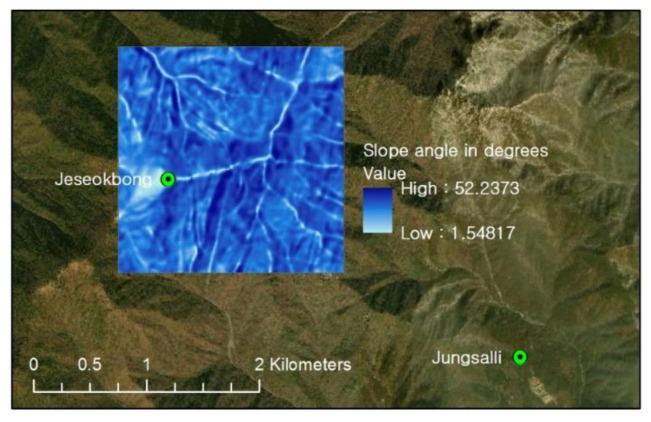
Slope angles of the analysis domain area.

Effective cohesion was set as zero as previous researches reported that effective cohesion has insignificant effects on slope stability analysis involving Korean weathered residual soil, and have thus been set as zero in multiple studies [40,41,42]. In addition, several overseas studies also set effective cohesion as zero for the conservative analysis of sandy soil slopes with low cohesion effects [43,44]. Laboratory tests for 17 different soil samples that had been collected inside and around the domain area proved that the area is characterized as a weathered sandy soil area with percent fines more or less 3%. Regarding the effective internal friction angle, the soil samples exhibited variation from 29° to 38°. Given that in the geological conditions of Korea the effective internal friction angle is a much less sensitive parameter for slope stability than an unsaturated soil stress state parameter (i.e., suctions stress) that is governed by subsurface hydrology [45], the effective internal friction angle was fixed to a mean value of 34.5° to focus on the effects of temporal variations of suction stress and spatial variations of slope angle. The range of slope angles of the study area was derived using a digital elevation model with a spatial resolution of a 10 m × 10 m cell grid, as shown in Figure 11.

## 4. Results and Discussion

### 4.1. Monitored Rainfall and Matric Suction Data

**Figure 12:**
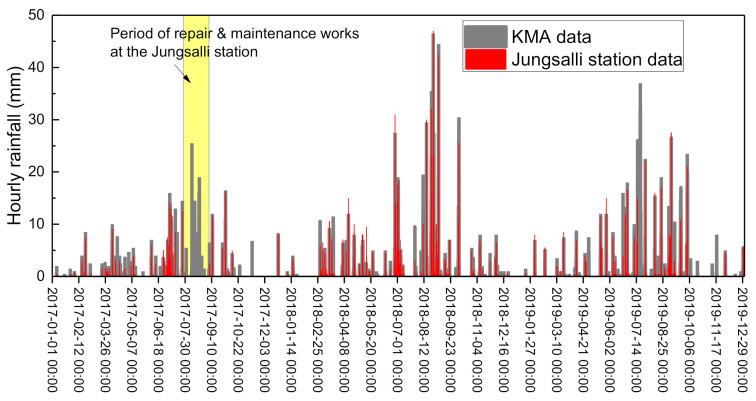
Comparison of the monitored rainfall data of the Jungsalli station and KMA.

The data from the Jungsalli station were used for the analysis domain area. This was because the rainfall data were measured and stored with stability at the Jungsalli station, and this data did not show significant deviations from those of the Jeseokbong station. To examine the reliability of the rainfall data collected by the rain gauges installed at the Jungsalli station, the data were compared with the rainfall data measured by a weather observatory (observatory name: Jirisan) of the Korea Meteorological Administration (KMA) in the vicinity of the analysis domain area. In contrast to the rain gauges of the monitoring station, which are located near the peak of Jirisan Mountain, the KMA weather observatory is located at the middle and lower parts of the mountain. Figure 12 illustrates a comparison between the rainfall data over a three-year period from 2017 to 2019 of the monitoring station and the KMA weather observatory with a fixed rainfall recording interval of one hour. As shown in the figure, the two sets of rainfall data were similar with no significant discrepancies, indicating that the rainfall data collected from the monitoring station rain gauges are highly reliable. However, rainfall data between late July and early September 2017 were not recorded as this period was dedicated to the replacement and maintenance of the communications system. Therefore, the rainfall data for this period referred to the data of the KMA weather observatory.

**Figure 13:**
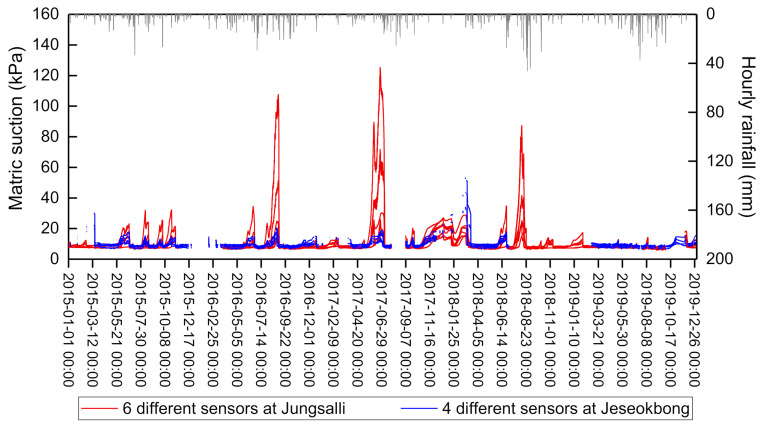
Monitored matric suction data of the Jeseokbong and Jungsalli stations.

Figure 13 shows the matric suction data measured by a total of 10 sensors of the Jeseokbong and Jungsalli stations from 2015 to 2019. As shown in the figure, the matric suction increases to a maximum of 120 kPa during extended dry seasons, and rapidly decreases to near zero upon rainfall. The matric suction measurement data of the Jeseokbong station were generally within the measurement range of the Jungsalli station. In addition, the increase in matric suction during dry seasons recorded by the Jeseokbong station was somewhat lower compared to the measurement results of the Jungsalli station. This is believed to be due to the Jeseokbong station being located at a higher altitude near the peak of the mountain, where the temperature is relative lower and the humidity relatively higher. In addition, most of the missing periods in the measurement data of the Jeseokbong station occurred during winter, when there is little rainfall. Considering the aforementioned findings, the measurement data of the Jungsalli station are deemed as a suitable substitute to replace missing data from the Jeseokbong station.

### 4.2. Temporal Evaluation of Suction Stress and Slope Stability Using Monitored Data

Landslides that occur in Korea are caused as rainfall infiltrates the ground surface during heavy rainfall and reduces the shear strength of the soil below a certain threshold, resulting in sliding. Such decreases in shear strength that lead to landslides are mainly due to an increase in soil saturation and the resulting decrease in unsaturated suction stress. Therefore, matric suction, an unsaturated property of natural slope soils, is measured in real time to enable the calculation of suction stress. This in turn enables the calculation of the factor of safety through infinite slope stability analysis.

**Figure 14:**
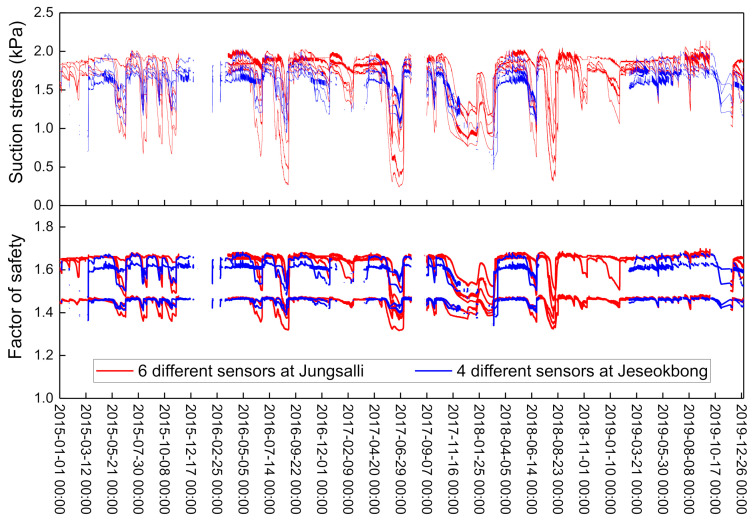
Suction stress and factor of safety calculation results of the Jeseokbong and Jungsalli stations.

The upper graph in Figure 14 shows the change in suction stress over a five-year period from 2015 to 2019, based on calculations using matric suction and Equation (3). The matric suction values measured by the monitoring stations and the van Genuchten [38] fitting coefficients (Table 5) that represent the unsaturated properties of the analysis domain area were applied to the equation. The calculated suction stress of the soil varied within the range of 0.2~2 kPa depending on rainfall. As previously described, it is possible to derive the factor of safety once the suction stress of natural slope soil is calculated.

The lower graph of Figure 14 shows the factor of safety values for a slope with an angle of 28°, which represents the slope angle of the exact points where the sensors are located. These were calculated through Equation (5) by applying the input data from Table 5. In Equation (5), the depth of the shear plane was set as 0.5 m or 1 m depending on the depth of each sensor. As shown in the figure, the variation in the slope factor of safety depended on the variation in the soil suction stress. At a depth of 0.5 m, the slope factor of safety varied within an approximate range between 1.7 and 1.4, and at a depth of 1 m, the slope factor of safety repeatedly increased and decreased within an approximate range between 1.45 and 1.32. Considering that landslide events were not reported nor were any traces of newly developed landslides found from satellite images during the monitoring period, one of the analysis results that the factor of safety value did not decrease to less than 1.0 during same period seems reasonable.

As a result, it is possible to evaluate slope stability according to real-time rainfall by monitoring the unsaturated properties of the slope soil. Furthermore, the evaluation results highlight the potential of the monitoring system for landslide forecasting. By establishing landslide prognosis thresholds, which are indices of how unstable a slope is, warnings can be issued depending on the calculated factor of safety value relative to the thresholds. Notably, as such landslide early warning methods based on real-time monitoring do not use prediction data, it will be crucial to determine a set of conservative landslide occurrence thresholds to ensure adequate lead time.

### 4.3. Spatial Classification of Landslide Hazard Using Monitored Data

**Figure 15:**
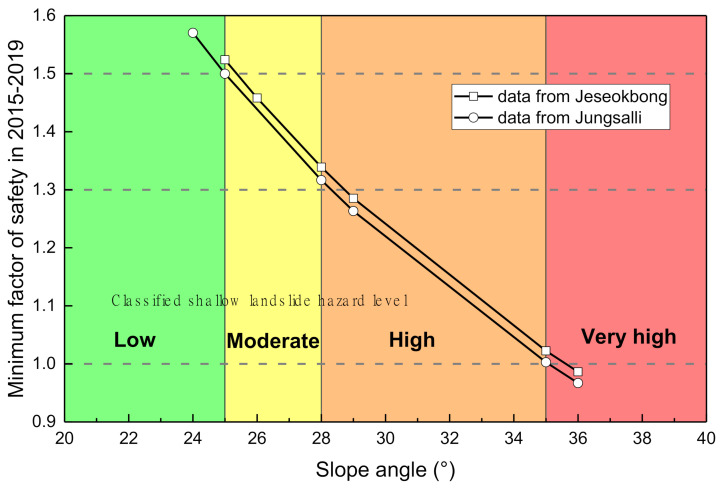
Minimum factor of safety values according to slope angle within the monitoring period and determination of landslide hazard levels.

Assuming near-homogeneous conditions in geotechnical and unsaturated soil properties within the analysis domain area, the slope angle (*β*) becomes the only spatially variable parameter for the infinite slope stability equation (Equation (5)) at a particular time. In other words, all slopes in the analysis domain area are characterized by the data in Table 5 and Figure 10, and thus it is possible to calculate the factor of safety according to slope angle for the monitoring period from 2015 to 2019. Therefore, it is possible to derive the minimum factor of safety value that represents the most unstable state within the monitoring period, which varies according to slope angle. Figure 15 shows the minimum factor of safety according to slope angle calculated using the five-year monitoring data of the Jeseokbong and Jungsalli stations: the minimum factor of safety decreases with increasing slope angle. Then, several thresholds were adopted to classify landslide hazard levels according to slope angle. In slope design, the factor of safety threshold is usually set as 1 or 1.3 to determine instability; in this study, a more conservative value of 1.5 was adopted as the threshold. Subsequently, if the minimum factor of safety during the long-term monitoring period of five years is greater than 1.5, it is considered as “Low” hazard level. “Moderate”, “High”, and “Very high” hazard levels were defined as values between 1.3 and 1.5, between 1.0 and 1.3, and less than 1.0, respectively. As a result, as illustrated in Figure 15, if the slope angle is less than 25°, the landslide hazard level is set as “Low”. “Moderate”, “High”, and “Very high” are applied if the slope angle is between 25° and 28°, between 28° and 35°, and greater than 35°, respectively.

**Figure 16:**
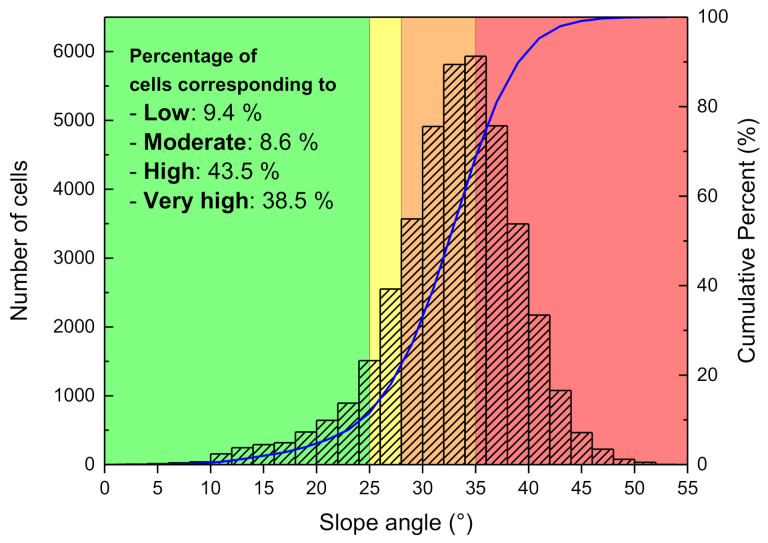
Slope angle distribution and landslide hazard levels of the analysis domain area.

**Figure 17:**
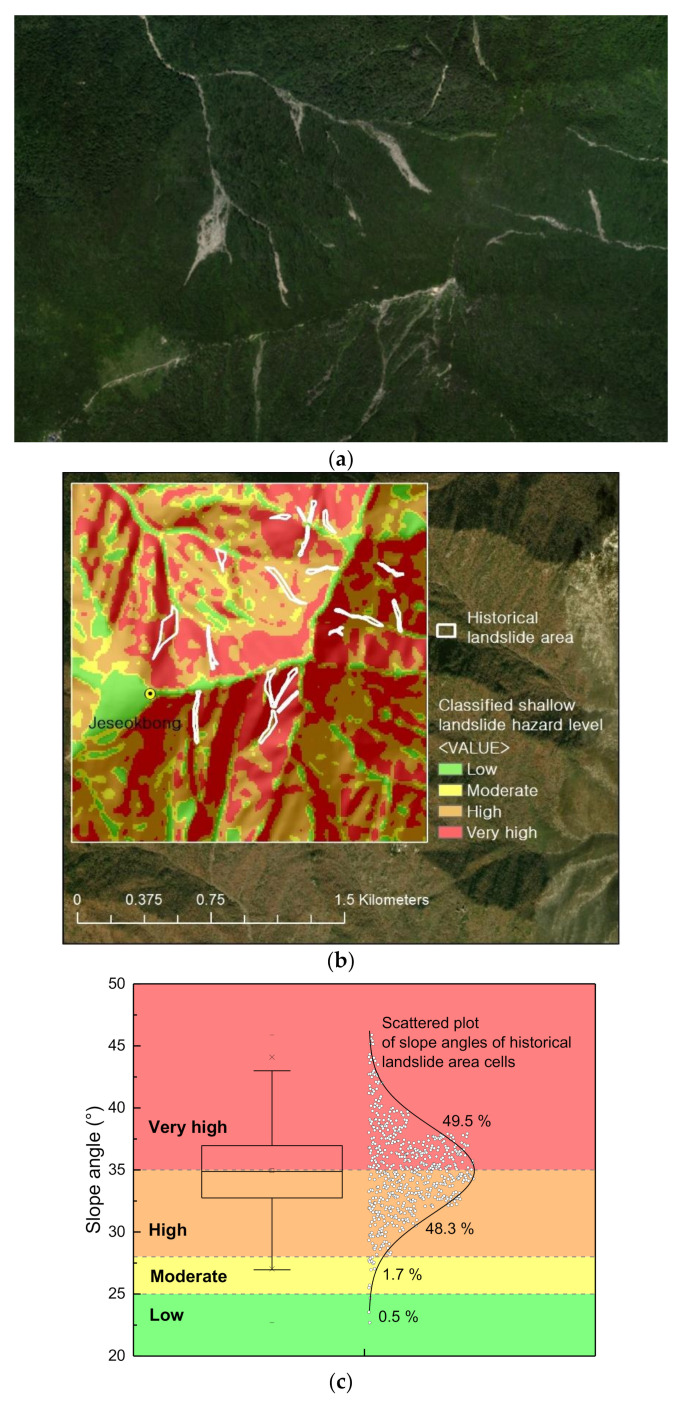
Evaluation results of the landslide hazard levels of the analysis domain area: (**a**) Historical landslide areas in the domain area (satellite image taken in 2018); (**b**) Spatially classified landslide hazard mapping; (**c**) Landslide hazard class distribution of the historical landslide areas.

In order to apply the aforementioned landslide hazard thresholds, a digital elevation model (DEM) with a resolution of 10 m was produced for the 2 km × 2 km analysis domain area, and thus a total of 40,000 grid cells constituted the analysis domain area. Figure 16 classifies the slope angle distributions of the grid cells constituting the analysis domain area. The area consists of rather steep terrain with an average slope angle of approximately 33°. In addition, 82% of all cells (the total area) were observed to fall under the high hazard levels of “High” and “Very high”. Therefore, it can be said that the study area is overall highly likely to landslide occurrences. Such evaluation results are supported by the many landslide scars that can be observed in satellite images of the study area (Figure 17a).

Figure 17b shows the spatial classification results of the analysis domain area according to the landslide hazard level. In this result, the polygon areas with white borders are areas with confirmed landslide occurrences according to a comparison study with satellite images by year. As based on infinite slope stability analysis, the landslide hazard classification results represent the predisposition of slopes to initiation of sliding. Therefore, for comparative analysis with landslide historical data, only areas that were clearly identified as source areas of landslides were included; flow channel areas resulting from erosion mechanisms exerted by fluidized sliding mass were excluded from the analysis. Figure 17c shows the slope distribution and hazard level classification results of a total of 683 cells that constitute the historical landslide areas. The average slope of the historical landslide areas was approximately 35°, and the box plot shows that half of the areas had slopes ranging approximately between 33° and 37°.

Moreover, approximately 98% of the historical landslide areas were confirmed to be classified with the “High” or “Very high” hazard levels. Considering that almost all historical landslide areas were located in areas with high landslide hazard levels, the proposed method for the spatial classification of landslide hazards based on long-term monitored data is expected to be effectively utilized as a reliable fundamental resource for decision making in disaster management policies and future land-use planning. However, it should be noted that the validity of the proposed spatial classification method is restricted to shallow depth planar landslide types. From a theoretical perspective, if the factor of safety is less than 1, this indicates that the shear stress has exceeded the shear strength, meaning there is a high chance that a slope failure or landslide has occurred.

However, landslides have yet to actually occur in a significant portion of areas under the “Very high” hazard level due to various factors: spatial uncertainty of soil engineering properties or unsaturated characteristics, including increased strength due to the effective cohesion of soil or root cohesion effects from vegetation, and exposure of bedrock or soil depths of less than 1 m due to the loss of soil in case of steep slope angles. The idealization of problems, simplification processes, and conservative designs are inevitable issues when applying modelling techniques for landslide prediction. Therefore, areas with high landslide hazard levels that have yet to experience landslides should not be considered as overestimated predictions, but rather as areas with a high potential for landslide occurrence that can be triggered by minute variables at any moment in the future.

## 5. Conclusions

An integrated landslide monitoring system, denominated as KIGAM-LAMOS, was developed to measure rainfall and the resulting unsaturated soil behaviors in real time that cause shallow landslides in natural slopes. Based on the long-term monitoring results, it was possible to quantitatively evaluate landslide hazards in the analysis domain area. These include temporal variations of suction stress in unsaturated soil and slope stability and spatial classifications of landslide hazard levels.

Monitoring stations of KIGAM-LAMOS were installed at 12 natural slopes across eight national parks in Korea. Each station had various sensors to measure rainfall and unsaturated soil properties in real time in addition to various equipment for the collection and transmission of the measurement data. The monitoring stations were installed in areas that exhibited representative geological conditions of Korea, namely those of granite, gneiss, and sedimentary rock lithology. In addition, a cloud server-based landslide monitoring operation program was developed to provide a user-friendly interface that allowed users to effectively store and manage the sizeable amounts of data being transferred from the multiple monitoring stations.

To test the potential of the landslide monitoring system for detecting and forecasting landslides, a 2 km × 2 km landslide-prone area around the Jeseokbong monitoring station in Jirisan National Park was set as the analysis domain area. Then, the analyses for landslide hazard evaluations based on infinite slope stability analysis were conducted by analyzing monitored data gathered over a five-year period from 2015 to 2019. The data missing from the Jeseokbong station were supplemented by data monitored at the nearby Jungsalli station. The time-series variations in the suction stress of slope soil layers could be calculated using the monitored matric suction data and unsaturated soil laboratory test data. The calculated suction stress data were subsequently used to calculate the factor of safety within the monitoring period. With this as a basis, the slope factor of safety according to real-time rainfall was calculated, which highlighted the temporal capability of the monitoring system for detecting landslide hazards. As such landslide detecting (or forecasting) methods based on real-time measurements do not use prediction data, it will be crucial to set multiple conservative thresholds to ensure adequate lead time.

By assuming that the analysis domain area has spatially uniform soil properties and unsaturated characteristics, the spatial classification analysis of landslide hazards was conducted using the long-term factor of safety calculation results. Upon comparing the historical landslide areas that occurred in the analysis domain area with the spatial classification results, it was found that almost all of the historical landslide areas were located in areas with high hazard levels. As such, the reliability of the proposed landslide hazard classification method was validated.

The present study covers the establishment of a landslide monitoring system and shows the beginning stage of its applications. We are sustaining our monitoring system to keep collecting the measurement data as well as future landslide events. Based on such cumulated dataset, we expect that even more valuable findings could be published in the future to contribute to landslide community.

Although KIGAM-LAMOS was verified in its effectiveness in landslide hazard evaluations, a monitoring-based shallow landslide management method cannot stand alone. This is mainly because its effectiveness is limited to the local area of sensing. Therefore, further studies should be directed towards the integration of the developed monitoring system with analytical methods that can cover regional-scale areas.

## Figures and Tables

**Table 1:** Key specifications of the sensors in KIGAM-LAMOS.

Sensor Specifications	Measurement Type (Model Code)				
	Volumetric Water Content/Temp. (Decagon 5TM)	Matric Suction (Decagon MPS-6)	Slope Displacement (NGI MV-5B)	Debris Flow (Sakada Denki EDZ-004)	Rainfall (Wedaen WDR-205)
Dimensions (L × W × H)	10 cm × 3.2 cm × 0.7 cm	9.6 cm × 3.5 cm × 1.5 cm	12 cm(φ) × 6.3 cm (H)	36 cm × 4.4 cm × 7.5 cm	Extenal: 40 cm (φ) × 52 cm (H) Internal: 20 cm (φ) × 52 cm (H)
Measurement range	0~100% VWC/ −40~60 °C	−40~60 °C	±5 deg	±25 mm	0.5 mm
Resolution	0.1 ε_a_/1 °C	0.1 °C	0.15% FS	-	1 mm/h
Measurement time	150 ms	150 ms	1.0 s	1.0 s	150 ms
Operating temperature	−40~60 °C	0~60 °C	−20~80 °C	−30~50 °C	0~80 °C

**Table 2:** Installation locations of the landslide monitoring stations in eight national parks.

Mountain National Park		Coordinate of Monitoring Station	
		X	Y
Seoraksan	Baekdamsa	128°22′17.67″ E	38°10′45.83″ N
	Huiungak	128°27′55.52″ E	38°07′56.82″ N
Juwangsan		129°06′58.75″ E	36°26′30.43″ N
Songnisan		127°49′49.71″ E	36°33′18.20″ N
Gyeryongsan		127°17′20.19″ E	36°19′54.68″ N
Deogyusan		127°44′47.15″ E	35°51′34.69″ N
Mudeungsan		126°59′35.70″ E	35°07′07.20″ N
Wolchulsan		126°41′10.56″ E	34°45′13.99″ N
Jirisan	Jeseokbong	127°43′17.55″ E	35°20′03.85″ N
	Jungbong	127°44′00.63″ E	35°20′32.71″ N
	Rotary Shelter	127°44′17.57″ E	35°19′35.96″ N
	Jungsalli	127°45′25.62″ E	35°19′18.42″ N

**Table 3:** Geological conditions of the landslide monitoring station locations.

Station Site	Geological Period	Geological Conditions
Seoraksan	Jurassic period of the Mesozoic Era	Daebo granites
	Cretaceous period of the Mesozoic Era	Bulguksa granites
	Jurassic period of the Mesozoic Era	Two mica granite
Jirisan		Mixed pegmatite gneiss
	Age unknown	Porphyroblastic gneiss
		Gneiss granitic gneiss
Songnisan	Cretaceous period of the Mesozoic Era	Gyeongsang alkali granite
Juwangsan	Cretaceous period of the Mesozoic Era	Red sandstone layer of the Gyeongsang Silla Group-Nakdong group
Mudeungsan	Cretaceous period of the Mesozoic Era	Mudeungsan quartz andesite
Deogyusan	Precambrian	Deogyusan layer of the Precambrian Weonnam group
Wolchulsan	the Cretaceous	Red feldspar granite
Gyeryongsan	Cretaceous period of the Mesozoic Era	Quartz porphyry

**Table 4:** Sensors installed in the landslide monitoring stations.

Station Site		Number of Sensors According to Measurement Type					Number of Data Logger
		Volumetric Water Content	Matric Suction	Slope Displacement	Debris-Flow Wire	Rainfall	
Seoraksan	Baekdamsa	12	12	2	3	1	1
	Huiungak	12	12	2	-	1	1
Juwangsan		12	12	2	-	1	1
Songnisan		18	18	2	-	1	1
Gyeryongsan		12	12	2	-	1	1
Deogyusan		18	18	2	-	1	1
Mudeungsan		18	18	2	-	1	1
Wolchulsan		18	18	2	-	1	1
Jirisan	Jeseokbong	8	8	2	3	1	1
	Jungbong	8	8	2	2	1	1
	Rotary Shelter	12	12	2	-	1	1
	Jungsalli	12	12	2	-	1	1
Total		160	160	24	8	12	12

**Table 5:** Representative geotechnical property data of the analysis domain area. * n/a: not applicable.

Soil-Water Characteristic Curve (van Genuchten [38])					Dry Unit Weight	Effective Cohesion	Effective Angle of Internal Friction	Percent Fines	Liquid Limit	Plastic Limit
*θ_s_*	*θ_r_*	*α*	*n*	*m*	*γ_d_* (kN/m^3^)	*c*′ (kPa)	*φ*′ (°)	(%)	*LL* (%)	*PL* (%)
0.4254	0.0949	0.2807	2.7585	0.6375	15.30	0	34.5	3	23.6	n/a *

## Data Availability

Available upon request.
